# Mechanical Properties and Microstructure of Binary In-Sn Alloys for Flexible Low Temperature Electronic Joints

**DOI:** 10.3390/ma15238321

**Published:** 2022-11-23

**Authors:** Jiye Zhou, Xin Fu Tan, Stuart D. McDonald, Kazuhiro Nogita

**Affiliations:** 1 Nihon Superior Centre for the Manufacture of Electronic Materials (NS CMEM), School of Mechanical and Mining Engineering, The University of Queensland, St. Lucia, QLD 4072, Australia; 2 Department of Applied Quantum Physics and Nuclear Engineering, Kyushu University, Fukuoka 819-0395, Japan

**Keywords:** lead-free solder, mechanical properties, low temperature electronic joints

## Abstract

This research evaluates the mechanical properties of a variety of binary In-Sn alloys as potential candidates for low temperature electronic joints. The tensile and hardness tests of as-cast In-5Sn, In-12.5Sn, In-25Sn, In-30Sn, In-35Sn, In-40Sn, In-50Sn, In-60Sn, In-80Sn (wt.%) were assessed at room temperature and compared to those of pure In and Sn. The ultimate tensile strength (UTS) increased from 4.2 MPa to 37.8 MPa with increasing tin content in the alloys under the testing condition of 18 mm/min and the results showed little difference under a lower strain rate (1.8 mm/min). Most compositions showed good ductility in tensile testing with an average of 40% elongation. A melting point range of 119.3 °C to 194.9 °C for tested alloys was measured using differential scanning calorimetry (DSC). The microstructure investigated by scanning electron microscopy (SEM) was discussed with respect to the mechanical properties and it has been found that the presence of the Sn-rich γ-InSn_4_ phase in the microstructure has a significant impact on mechanical properties. The fundamental data from this study can be used for the development of new low temperature In-Sn alloys.

## 1. Introduction

The high demand for environmentally friendly and reliable products has driven research on developing lead-free solders with better mechanical properties. Accordingly, environmental-friendly Sn-based lead-free solders such as Sn-Cu (227 °C), Sn-Ag (221 °C), Sn-Ag-Cu (217 °C), Sn-Zn (198 °C) and their composites have been studied and developed for replacing the conventional Sn-Pb (183 °C) solders to fulfill the restrictions on hazardous substances by European regulatory bodies [1,2,3,4]. However, the relatively high melting point of these alloys is associated with increased energy consumption and creates difficulties when soldering temperature-sensitive components that may be present in standard optoelectronic integrated circuits (OEICs) [4,5,6], magnetic resonance imaging (MRI) scanners [7] and biological electronic devices [8]. Solders from the In-Sn alloy system can offer potential advantages as joining materials for these applications as they have relatively low melting points and can help relieve mechanical stresses inside the electronic package due to their high ductility [9,10,11]. Moreover, for specific aerospace and low-temperature applications Indium-based solder alloys are considered to have better reliability under extended thermal cycles as they have been proven to retain good ductility even under cryogenic conditions [12,13].

As electronic devices may experience vibration and impact loading during use, sufficient mechanical integrity of the solder alloys is required to guarantee reliability. Indium is an extremely soft metal with a reported ultimate tensile strength (UTS) of 1.6 MPa and 60% average elongation (9 × 10^−5^ s^−1^ strain rate) at room temperature [10]. As the β-In_3_Sn phase in the Indium-Tin system has a body-centered tetragonal crystal structure with the same space group I4/mmm as pure Indium, high ductility can also be expected in this In-rich phase. In comparison, the γ-InSn_4_ phase has a hexagonal crystal structure which contributes different physical properties to the In-Sn system. It has been proposed that recrystallisation within the γ-InSn_4_ phase might occur during shear testing and result in strain softening [5]. The eutectic alloy In-48Sn has attracted attention due to it having the lowest melting point (118 °C) in the In-Sn system [14,15,16,17]. An average UTS of 13 MPa with 34% elongation for In-48Sn solder at room temperature has been reported in early studies [11,18], and failure by intergranular cracking and cleavage between the β and γ phases has been reported [18]. A higher elongation of 83% for In-48Sn in shear testing has been recorded, with the higher elongation and longer isothermal fatigue life being associated with a uniform superplastic microstructure [2].

Recently, studies revealed that the volume fraction of the β-In_3_Sn phase and γ-InSn_4_ could affect the superconductivity properties in In-Sn solders [19,20]. However, due to the difficulty of sample preparation of Indium-based alloys (due to the extreme softness of Indium) and the relatively high cost, there are few fundamental studies for these novel solder alloys. To fill the knowledge gap and investigate the mechanical properties in this ultra-low temperature binary solder system, the testing for UTS, elongation, and Vickers hardness were carried out for a range of compositions in the In-Sn binary system. The mechanical properties have been discussed in relation to the volume fraction of β-In_3_Sn phase and γ-InSn_4_ in the microstructure and thermal properties.

## 2. Materials and Methods

### 2.1. Materials and Samples Preparation

**Figure 1:**
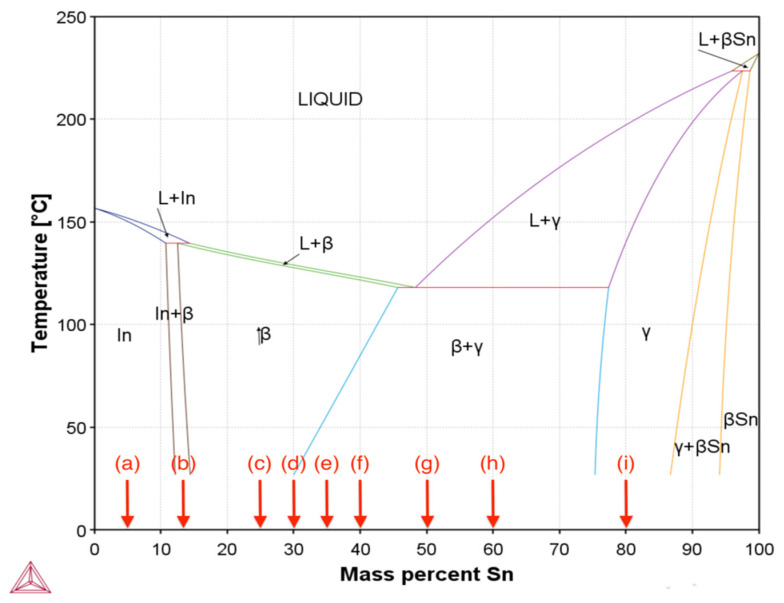
In-Sn binary phase diagram calculated based on Thermo-calc 2022a [22] database (TCSLD4: Solder Alloy v4.1) with binary alloy compositions studied in this work, the Sn content increases from (a) 5 wt% to (i) 80 wt% (pure Sn and In were investigated in addition to these compositions).

**Figure 2:**
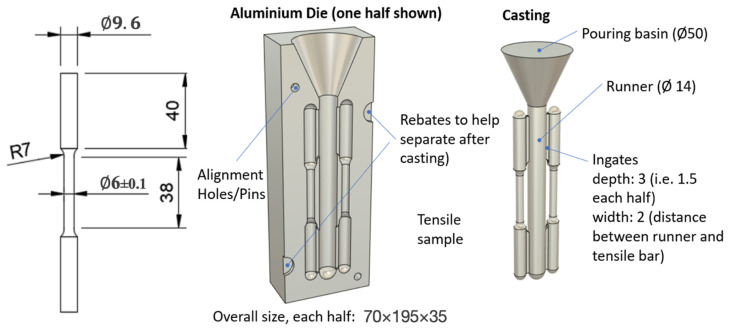
Schematic of the tensile bar and aluminum mold and resulting casting (all dimensions in mm).

To obtain tensile bars for testing, pure indium (99.995%) ingots (supplied by Nihon Superior) and tin (99.99%) ingots (supplied by Northern Smelters, Woodridge, QLD, Australia) were alloyed to obtain a range of In-Sn alloys. As shown in the In-Sn phase diagram (Figure 1), 9 different alloys as well as pure Indium and Tin were selected for the testing. Weighed ingots were melted using graphite crucibles in an electric resistance furnace at temperatures 220 °C above their equilibrium melting point for one hour (e.g., the pure Indium samples were melted at 376 °C). Any dross formation on the surface of the liquid metal was mechanically removed and stirring was performed every fifteen minutes to achieve a homogenous melt. As shown in Figure 2, samples were cast into a boron-nitride coated aluminum mold with the geometry of the tensile bars compliant with the ASTM E8/E8M standard [21] and then cooled in air to make tensile bars for testing. As the Indium based samples are soft and fragile, to avoid bending due to external forces, tensile bars were supported with a custom 3D printed jig, conformal to the bar geometry, while being cut from the body of the main casting.

### 2.2. Mechanical Properties Test

The tensile testing was performed in an Instron (model 5584) machine with a 10 kN load cell for In-80Sn samples and a 1 kN load cell for remaining compositions. Two different crosshead displacement rates, 1.8 mm/min and 18 mm/min, were selected for comparison and a video extensometer was used to measure the strain. A 30 mm gauge length was used for the video extensometer and the diameter of the reduced section of the tensile bar was 6 mm. For each composition, at least 10 tensile bars were tested. As casting defects are well recognised to be associated with reduced elongation [23], only the three samples with the longest elongation were selected for reporting for each alloy, under the assumption these are the most likely to be defect-free.

The Vickers hardness (HV) test was performed using a Struers Vickers Micro-Hardness Machine with 980.7 mN test load and 12 s dwell time. The reported results are the average of six individual indents on each sample.

### 2.3. Thermal Properties Characterisation

The thermal properties of the In-Sn solder alloys were tested by a Differential Scanning Calorimetry (DSC) machine (TA Discovery DSC 2500). Samples of 10 ± 3 mg small pieces were packaged into Tzero Aluminum pans and lids (Waters Australia Pty Ltd., Rydalmere, NSW, Australia) and heated up from the standby temperature of the machine (40 °C) to a temperature above its melting point, then cooled to 20 °C. The ramp rate was set to 10 °C/min and nitrogen gas flow was set to 50 mL/min to protect the samples from oxidation. The measurement was conducted twice for each composition (totally 18 samples) to ensure repeatability.

### 2.4. Microstructural Analysis

Metallographic preparation of Indium based alloys is difficult due to the softness of this material. To avoid the embedding of polishing media in the soft β phase, an ion milling machine (Hitachi IM 4000 Plus Ion mill, Hitachi, Japan) was used to obtain a clean surface finish instead of conventional grinding and polishing processes. The cross section of each sample was cut with a surgical knife before ion milling for 15 min under a 3 kV accelerating voltage with 120 μA ion beam current and protected under a 0.11 mL/min argon gas flow. The microstructure images of prepared samples were captured using a scanning electron microscopy (SEM) (Hitachi TM3030) under backscatter electron (BSE) mode at 15 kV accelerating voltage. The energy dispersive spectroscopy (EDS) analysis for elemental mapping was carried out using Bruker Quantax 70 software.

## 3. Results and Discussion

### 3.1. Thermal Properties and Microstructure Formation in In-Sn Solder Alloys

#### 3.1.1. Melting Point Characterisation

The detailed endothermic peak temperature and exothermic peak temperatures were further analysed by the TA DSC software and are summarised in Table 1 and Table 2, respectively. DSC curves of In-Sn solder alloys are shown in Figure S1 (Supplementary Materials). As the tin content increases from 5 wt% to 50 wt%, a decrease in melting point from 152.1 °C to 119.3 °C is evident from Table 1.

In the hypoeutectic In-5Sn alloy there is only one reaction, namely the solidification of In-rich solid solution. In the In-12.5Sn curve, two reactions are visible, the first being solidification of In-rich solid solution which is quickly followed by a second peak at 138.6 °C which likely involves the formation of β-In_3_Sn, either from a peritectic reaction, as per the equilibrium phase diagram, or direct solidification of β-In_3_Sn in a non-equilibrium reaction. The solidification of the remaining hypoeutectic alloys was associated with only one peak on the DSC curve and solidification is likely to have involved only the β-In_3_Sn phase which, from Figure 1, forms over a very narrow temperature interval. There was no eutectic reaction evident during solidification of any of the hypoeutectic alloys. In contrast, the near-eutectic alloy displayed only one reaction at a low temperature consistent with the eutectic reaction, and on heating, the melting point of this alloy was 119.3 °C compared to a 118 °C melting point at the eutectic composition (In-48Sn) reported in an earlier study [17].

In-60Sn has the most phase transitions detected by DSC analysis (Figure S1h). The endothermic Peak I (119.2 °C) in the heating curve represented the β-In_3_Sn reaching a temperature at which the eutectic started to melt resulting in remaining liquid + γ-InSn_4_; while the two reactions Peak I (142.7 °C) and Peak II (116.9 °C) on the cooling curve represented the primary γ-InSn_4_ phase formation followed by a large fraction of eutectic β-In_3_Sn + γ-InSn_4_.

As shown in Table 1, the highest melting point of 194.9 °C was found in In-80Sn with an onset temperature of 175.3 °C, during solidification. Despite the equilibrium phase diagram of Figure 1 prediction that no eutectic should form in the In-80Sn alloy, a small amount of eutectic solidification occurs at 117.2 °C (compared to 116.9 °C in In-60Sn). This is consistent with predictions from a Scheil model of solidification as shown in Figure S2i (Supplementary Materials). On heating there is no reaction at this low temperature in the In-80Sn alloy indicating the kinetics of diffusion are sufficient that the sample is likely 100% γ phase prior to reaching the eutectic temperature. There are two remaining peaks that occur at very low temperatures in both the In-40Sn (26.2 °C) and In-60Sn (48.5 °C) samples and it is hypothesized that these are likely to relate to the β phase being able to dissolve less Sn at these low temperatures and some precipitation of γ phase occurring.

#### 3.1.2. Thermodynamics and Phase Formation

The enthalpy of fusion was calculated by the endothermic peak area with the DSC machine software as shown in Table 1. The calculation method is shown in Equation (1) [24].

(1) $$\Delta H=\Delta H_{cal}*\frac{A*m_{cal}}{A_{cal}*m}$$

where ∆*H* and ∆*H_cal_* are the enthalpy of fusion of the sample and calibration material, respectively (unit: J/g); *A* and *A_cal_* are the endothermic peak areas from the sample and calibration material, respectively; *m* and *m_cal_* are the mass of the sample and the calibration material, respectively.

Because of the constant pressure scenario during DSC testing, the relationship between enthalpy Δ*H_fusion_* and internal energy Δ*E* can be described by Equation (3) taking note of the first law of thermodynamics, as shown in Equation (2).

(2) ΔE=Q+W

(3) ΔH=ΔE+P∗ΔV

where Δ*E* is the change in internal energy, *Q* is the heat absorbed during fusion while *Q* = ∆*H* under constant pressure; Work *W* = *P*∗Δ*V* under constant pressure. Additionally, due to the relatively low volume change in the solid-liquid reaction, the enthalpy of fusion can indicate the change in internal energy of the reaction.

As shown in Table 1, the In-Sn solder alloys have an enthalpy of fusion in the range of 21.2 J/g to 47.8 J/g. Compared to the samples with 5 wt%Sn to 60 wt%Sn, there is a significant jump of enthalpy in In-80Sn which means the solid-liquid phase transformation in In-80Sn requires higher energy than other compositions. In addition, considering that In-80Sn has a significant volume fraction of γ-InSn_4_ phase, we can conclude that the formation of γ-InSn_4_ is associated with more heat and requires more energy than the β-In_3_Sn phase. The lowest enthalpy of 21.2 J/g can be found in In-40Sn; however, the difference in enthalpy among alloys with 25%wt to 40%wt Sn content is quite small due to the dominance of the β-In_3_Sn phase.

The volume fraction of phases in the In-Sn alloys during solidification, as calculated by the Scheil equation (via Thermo-calc) is shown in Figure S2. At 5 wt% Sn, a simple Indium solid solution forms and at 12.5 wt% Sn, this phase is joined by β-In_3_Sn. For all other hypoeutectic samples, β-In_3_Sn is the only phase predicted to form. The alloys with 50, 60 and 80 wt% Sn are predicted to have increasing amounts of primary γ-InSn_4_ phase followed by the remainder solidification of a eutectic mixture of β-In_3_Sn and γ-InSn_4_. These solidification pathways predicted by Scheil assumptions are in good agreement with the DSC results. However, they do not account for the solid-state precipitation of phases that can occur due to the changing solubility as the temperature decreases below the eutectic temperature and the relatively high homologous temperature of this alloy system at room temperature.

Despite the constant and relatively low ramp rate in DSC analysis, the DSC results show that non-equilibrium solidification occurs during cooling, as evidenced, for example, by the presence of a eutectic reaction in the In-80Sn alloy. However, on heating, the reactions appear to be closer to those expected from the equilibrium phase diagram. Notably, compared to other compositions, exothermic peaks can be found at near room temperature in both In-40Sn (26.2 °C) and In-60Sn (48.5 °C) (Table 2) which are neither predicted by the equilibrium phase diagram (Figure 1) or in Scheil volume fraction prediction (Figure S2f,h). These peaks may relate to the volume fraction change between β-In_3_Sn and γ-InSn_4_. During the cooling process, the microstructure continually adjusts by inter-diffusion of In and Sn atoms, with an increasing volume fraction of γ-InSn_4_ phase at the room temperature. In this case, the γ-InSn_4_ will either reprecipitate from the β-In_3_Sn phase at room temperature or the grain size of existing phases will adjust to reach an equilibrium. Compared to the In-35Sn and In-30Sn, the In-40Sn has more metastable saturated β-In_3_Sn on cooling, which might explain why the low temperature reaction is detected in this alloy. When it comes to In-60Sn, there is around 40% γ-InSn_4_ present in the microstructure before the β-In_3_Sn starts to form, as displayed in Figure S2h. The volume fraction between the γ-InSn_4_ and β-In_3_Sn suddenly changes as the eutectic reaction occurs when the temperature crosses the phase boundary which occurs at 116.9 °C, the predicted volume fraction of β-In_3_Sn increases to 55% in the microstructure. The large volume fraction of β phase in this alloy also experience a change in the solubility of Sn on cooling which may be correlated with the low temperature reaction detected on the cooling curve. Despite this, it remains unclear why the near eutectic alloy, In-50Sn, which will also contain a large fraction of β, does not display this low temperature reaction.

### 3.2. Mechanical Properties

**Figure 3:**
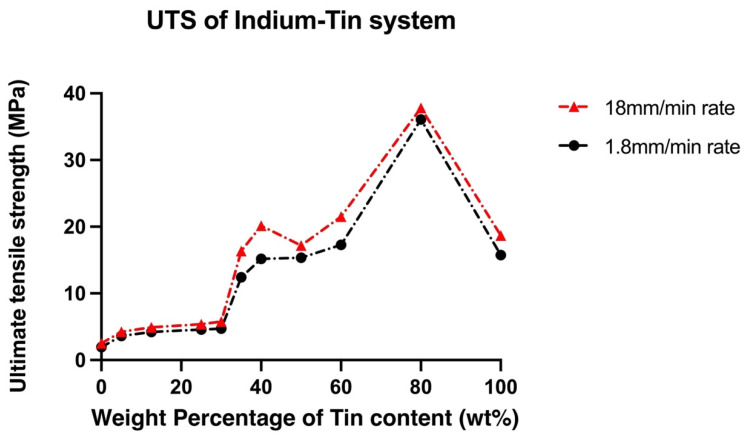
Ultimate tensile strength compared to tin content in In-Sn solder alloys under two strain rates.

**Figure 4:**
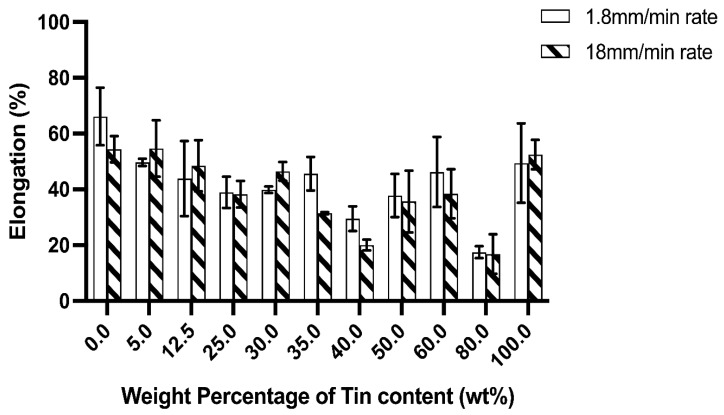
Elongation to failure compared to tin content in In-Sn solder alloys under two strain rates.

**Figure 5:**
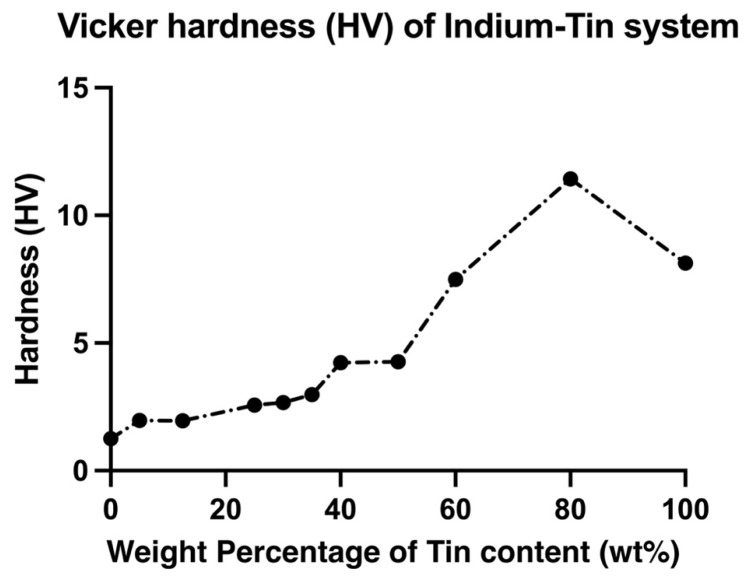
Average Vickers hardness of indium-tin solder alloys.

The change in UTS and elongation of In-Sn alloys with increasing tin content is plotted in Figure 3 and Figure 4. The mechanical properties of the In-Sn alloy studied in this work are summarised in Table S1 (Supplementary Materials). As the tin content was increased from 5 wt% to 60 wt% in the In-Sn alloys, there was a gradual rise in the UTS from 3.6 MPa to 17.3 MPa and 4.2 MPa to 21.5 MPa under different strain rates (as shown in Table S1). The UTS substantially increased to 36.1 MPa when the Sn content was increased to 80% which is even higher than the 15.8 MPa in pure tin; however, the elongation also decreased to 17.5% from 49.5% in pure tin. The elongation for compositions with 5 wt% to 60 wt% Sn content was in the range of 29.5% to 49.7% and 20% to 54.6% under two strain rates indicating that the ductility benefited from the presence of the β-In3Sn phase. Furthermore, the 37.8% elongation of In-50Sn closely matches the results previously obtained for the In-48Sn eutectic alloy in other studies, as shown in Table 3 [11,18]. The Vicker’s hardness of In-Sn alloys, plotted in Figure 5, closely matches the trends of the UTS data displayed in Figure 3. The highest hardness value, 11.43 Hv, was detected in In-80Sn, although it is noted that this is still lower than the 14.4 Hv associated with the Sn-3.8 wt%Ag-0.7 wt%Cu SAC solder alloy [25].

#### 3.2.1. Fracture Characterisation

**Figure 6:**
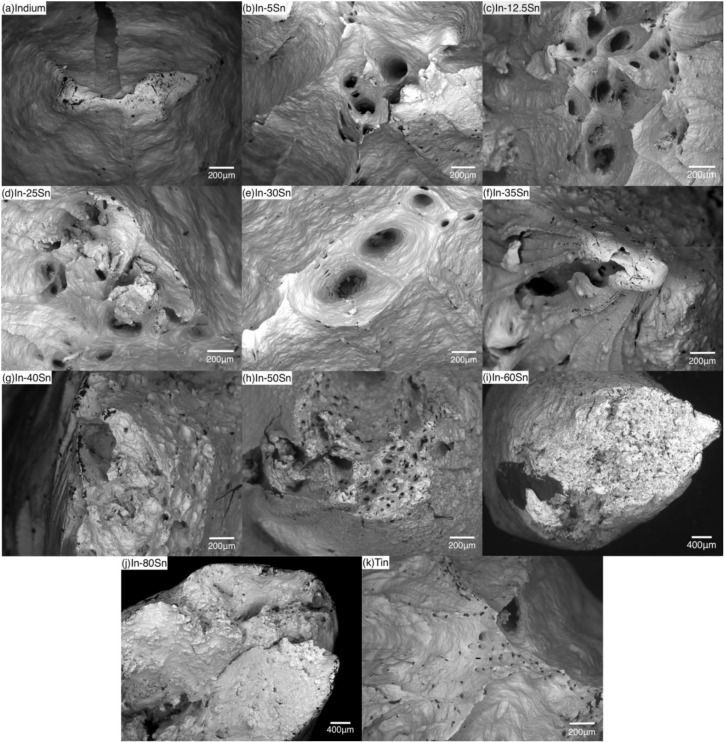
(**a**–**k**) Representative top view fracture surfaces of tested samples from 0 wt% Sn content to 100 wt% Sn content, where Figure (**i**) In-60Sn and (**j**) In-80Sn are under ×40 magnification and the rest of images are under ×100 magnification.

The top views of fracture surfaces are shown in Figure 6. The fracture surface of pure Indium shows a chisel/cone shape after the tensile testing (Figure 6a), consistent with the highly ductile failure mechanism. For the In-Sn alloys, the lower Sn contents were associated with a narrower neck, which increased in size and became flatter with an increasing of the Sn content. The increase in fracture surface cross-sectional area required different magnification imaging for the In-60Sn and In-80Sn samples. As shown in Figure 6b,h and Figure 6k, pores can be detected in the In-Sn alloys as well as the pure tin which correspond to the ductile failure mechanisms involving void growth and coalescence after extensive plastic deformation during the tensile testing [26]. The extent of ductile fracture is influenced by the volume fraction of the γ-InSn_4_ phase. As shown in Figure 6i,j, the fracture surfaces of In-60Sn and In-80Sn show flatter and larger fracture surface areas which are similar to the shear fracture in single crystals [27]. Therefore, the In-60Sn and In-80Sn present a more moderate ductile fracture compared to other compositions.

#### 3.2.2. Strain Rate Effect

The relationship between strain rate and UTS can be represented by the constitutive equation [5,28]

(4) $$\dot{\varepsilon }=A\sigma_{uts}{}^{n}\exp\left(-\frac{Q}{RT}\right)$$

where *A* is pre-exponential factor, which is a constant, *Q* is activation energy specific to the dominant deformation mechanism and *R* is the universal gas constant; $\dot{\varepsilon }$ is the strain rate and *n* is the stress exponent. As shown in Figure 6i,j, the fracture surfaces of In-60Sn and In-80Sn show a similar shape to the fracture caused by the dislocation slip of zinc [27]. This may support a deformation mechanism in higher Sn content alloys that is dislocation dominated. Notably, as mentioned above, the oversaturated Sn in β-In_3_Sn will affect the atomic density in the system; thus, the direction of dislocation movement will vary which results in a different shape of the fracture surfaces. However, as the deformation mechanism can also be varied under different strain rates and different phases, further research is needed.

Under the higher strain rate, the dislocation density is proposed to increase to accommodate the imposed strain rate [29]; as the dislocations move along the most-densely packed directions they may avoid the interaction of other dislocations, or in other words, there is not enough time for other dislocations to glide through [30]. Thus, the UTS value under higher strain rates (red line) shifts to higher values compared to the value under lower strain rates (black line), as shown in Figure 3. Compared to the UTS results, there is no uniform trend of increased or decreased elongation with higher strain rates (Figure 4), and, in general, all the In-Sn alloys showed relatively good elongation to failure.

#### 3.2.3. Microstructure

As shown in Figure 3, the UTS increases significantly as the amount of γ phase in the microstructure increases. Comparing In-80Sn (near 100% γ) and In-25Sn (100% β), it is apparent that the γ-InSn_4_ is relatively harder and less ductile while β-In_3_Sn is a soft and exceptionally ductile phase. For this reason, the volume fraction of phases is expected to have a close relationship to the mechanical properties. An average of 15 MPa UTS with a 40% elongation can be achieved in alloys with between 35 wt% Sn and 60 wt% Sn content which have a combination of both γ and β phases in the microstructure. Compared to these alloys, In-80Sn has an increase in the UTS but a decrease in elongation. Notably, compared to the In-40Sn, there was an increasing elongation in both In-50Sn and In-60Sn, which may have arisen due to strain softening brought about by the γ-InSn_4_ recrystallisation during the testing, which also resulted in a decrease of UTS in the In-50Sn alloy. To investigate the recrystallisation, high voltage—transmission electron microscopy (HV-TEM) with in-situ mechanical testing is proposed for a future study.

### 3.3. Microstructural Characterisation

**Figure 7:**
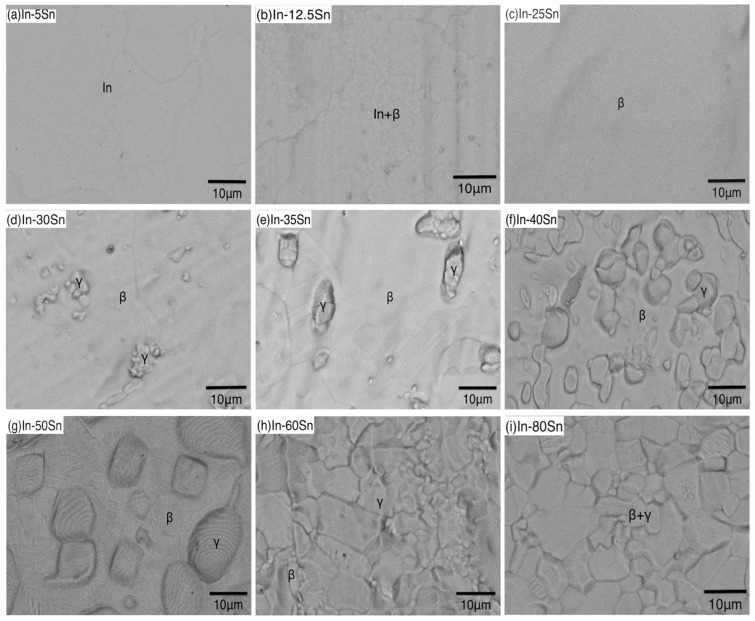
Microstructure of In-Sn alloys with increasing of tin content from (**a**–**i**).

The microstructures of In-Sn alloys studied in this work are shown in Figure 7 and related EDX mappings are displayed in Figure S3 (Supplementary Materials). The In-5Sn shows a clear microstructure dominated by indium, and grain boundaries can be easily detected in Figure 7a. The microstructure of In-12.5Sn shows a background with small particles scattered throughout and by referring to the calculated volume fraction in Figure S2b, these particles are likely to be indium. When the Sn content increases to 25 wt%, the alloy microstructure consists of 100% β-In_3_Sn with no second phase visible (Figure 7c). The γ phase is present in the microstructure when the Sn content is increased to 30 wt% and the volume fraction and grain size of the γ phase increased correspondingly with the increases in Sn concentration. Compared to the lamellar microstructure in the eutectic regions in other solder alloys, the microstructure showed irregular and island like grains in In-30Sn, In-35Sn, In-40Sn and In-50Sn (Figure 7d to g). Furthermore, as shown in Figure 7h, the γ phase dominated the microstructure when the tin content increased to 60 wt% and only small areas of β-In_3_Sn can be detected, consistent with the predicted volume fraction in Figure S2h; however, it was hard to distinguish the two phases by morphology alone. The γ phase showed irregular polygonal grains in In-80Sn (Figure 7i), with a high density of grain boundaries compared to the β phase shown in Figure 7c; the smaller grain size combined with the inherent properties of the γ phase, result in this composition having the highest UTS among all alloys tested.

## 4. Conclusions

The mechanical properties, thermal properties and microstructure of In-Sn solder alloys have been investigated in this study. Based on the results shown above, the following conclusions can be drawn:

- Different volume fractions of β and γ phases in In-Sn alloys had a significant effect on the mechanical properties; as compared to the β phase, the island-like γ phase is harder and less ductile. The increase of Sn content in the In-Sn alloys that were tested was associated with an increase in the ultimate tensile strength and hardness, and the highest UTS of 37.8 MPa has been measured in a composition of In-80Sn. Furthermore, ductile fracture was observed in all samples and good elongation was measured in most compositions.
- The DSC results showed a melting point range of 119.3 °C to 194.9 °C which are relatively low for solder materials. The calculated enthalpy values revealed that the formation of γ-InSn_4_ is associated with more heat and requires more energy than the β-In_3_Sn phase.
- In-Sn alloys have numerous applications as solder alloys for low-temperature processing and low temperature applications, especially where high ductility is required. The data from this study provides a good foundation for the further research into these alloys and gives some confidence on the expected mechanical properties. However, more mechanical property testing under cryogenic conditions as well as characterising the thermal cycling stability are required.

## Figures and Tables

**Table 1:** Thermal properties of In-Sn solder alloys during heating.

Test Sample	Onset temperature (°C)	Peak I (°C)	Peak II (°C)	Enthalpy ΔH (J/g)
In-5Sn	151.2	152.1	-	27.3
In-12.5Sn	141.8	143.5	-	25.4
In-25Sn	131.8	132.9	-	23.0
In-30Sn	128.4	129.2	-	23.2
In-35Sn	125.5	126.7	-	22.3
In-40Sn	121.7	123.0	-	21.2
In-50Sn	118.7	119.3	-	25.2
In-60Sn	118.6	119.2	150.7	28.9
In-80Sn	175.3	194.9	-	47.8

**Table 2:** Exothermic peak temperature of In-Sn solder alloys during cooling.

Test Sample	Peak I (°C)	Peak II (°C)	Peak III (°C)
In-5Sn	149.7	-	-
In-12.5Sn	141.1	138.6	-
In-25Sn	130.3	-	-
In-30Sn	126.6	-	-
In-35Sn	123.3	-	-
In-40Sn	120.7	26.2	-
In-50Sn	116.1	-	-
In-60Sn	142.7	116.9	48.5
In-80Sn	186.6	117.2	-

**Table 3:** Ultimate tensile strength and elongation of In-48Sn in previous studies.

Test Sample	Strain Rate	UTS (MPa)	Elongation (%)
In-48Sn	2.65 mm/min	13	34 [11]
	5 × 10^−4^ s^−1^	10.5	32 [18]

## Data Availability

The data presented in this study are openly available in the manuscript and the Supplementary Materials.

## Supplementary Materials

The following supporting information can be downloaded at: https://www.mdpi.com/article/10.3390/ma15238321/s1, Figure S1: DSC curves of In-Sn solder alloys with the increasing of tin content from (a) to (i). Table S1: Summary of mechanical properties (average) in In-Sn solder alloys (subscript indicates crosshead speed in mm/min). Figure S2: Volume fraction of phases in the In-Sn alloys during solidification calculated by Scheil equation (based on Thermo-Calc 2022a TCSLD4: Solder Alloys v4.1 database [22]) with increasing tin content from (a) to (i). The volume fraction of phases under the equilibrium condition is also plotted for comparison. Figure S3: Microstructure and EDX mappings of In-Sn solder alloys with increasing tin content from (a) to (i).
