# Supplementary material for: Mechanical Properties and Microstructure of Binary In-Sn Alloys for Flexible Low Temperature Electronic Joints
Source: Materials (Basel). 2022 Nov 23;15(23):8321. doi: 10.3390/ma15238321 (PMC9740450; doi:10.3390/ma15238321)
Supplement: Supplementary file 1 [file materials-15-08321-s001.zip › materials-2036811-supplementary.pdf]

materials

MDPI

Supplementary Material

Mechanical Properties and Microstructure of Binary In-Sn Alloys for Flexible Low Temperature Electronic Joints

Jiye Zhou [1], Xin Fu Tan [1,2], Stuart D. McDonald [1] and Kazuhiro Nogita [1,*]

[1] Nihon Superior Centre for the Manufacture of Electronic Materials (NS CMEM), School of Mechanical and Mining Engineering, The University of Queensland, St. Lucia, QLD 4072, Australia; jiye.zhou@uq.edu.au (J.Z.); xin.tan@uq.edu.au (X.F.T.); s.mcdonald1@uq.edu.au (S.D.M.)

[2] Department of Applied Quantum Physics and Nuclear Engineering, Kyushu University, Fukuoka 819-0395, Japan

* Correspondence: k.nogita@uq.edu.au; Tel.: +61-7-3365-3919

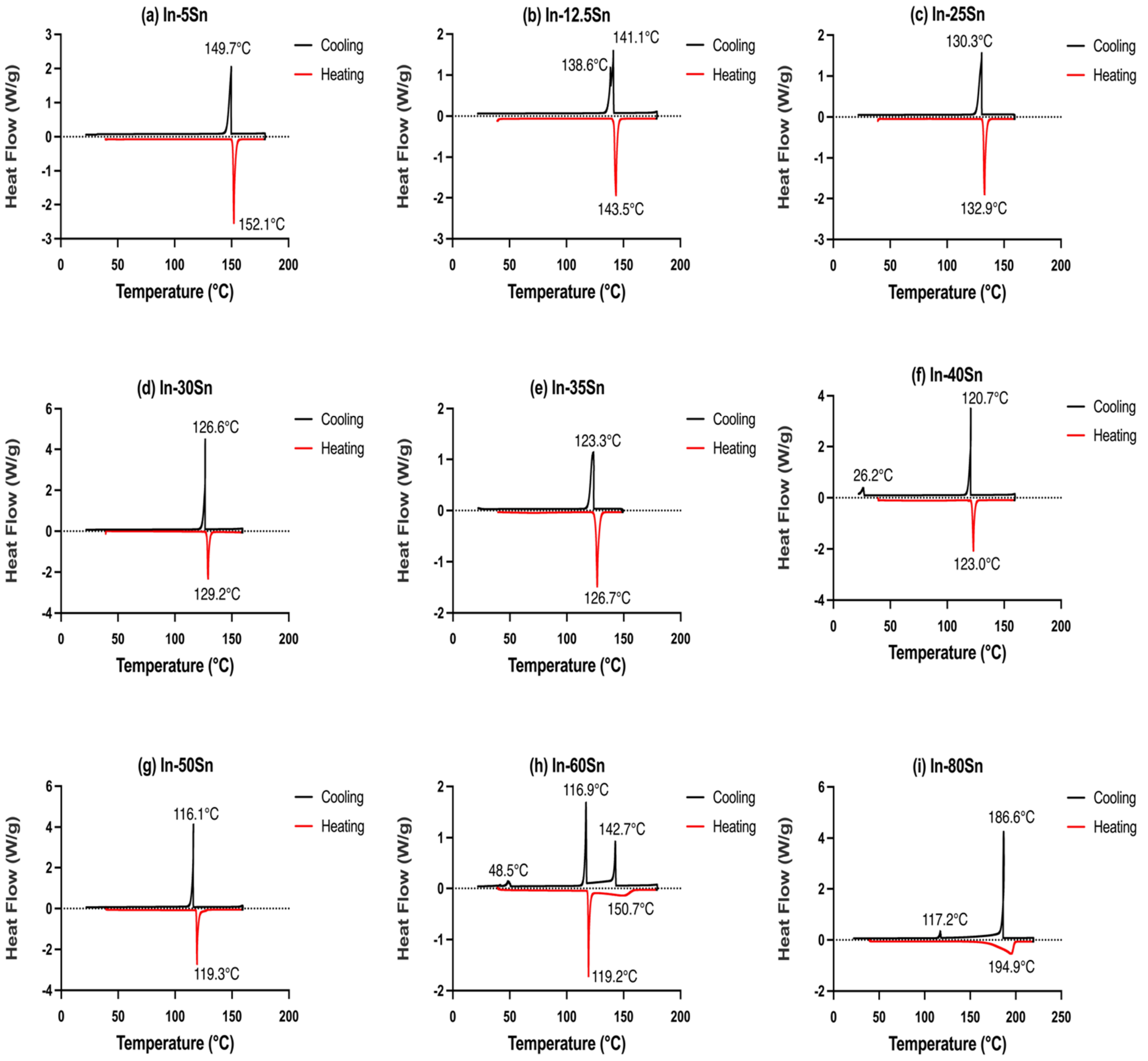

Figure S1. DSC curves of In-Sn solder alloys with the increasing of tin content from (**a**) to (**i**).

Table S1. Summary of mechanical properties (average) in In-Sn solder alloys (subscript indicates crosshead speed in mm/min).

**Test sample**	**$UTS_{1.8}$ (MPa)**	**UTS_{18} (MPa)**	**$Elongation_{1.8}$ (%)**	**$Elongation_{18}$ (%)**	**Hardness (HV)**
Indium	1.9 ± 0.1	2.5 ± 0.1	66.1 ± 10.3	54.4 ± 4.7	1.26 ± 0.02
In-5Sn	3.6 ± 0.1	4.2 ± 0.3	49.7 ± 1.3	54.6 ± 10.1	1.96 ± 0.04
In-12.5Sn	4.2 ± 0.2	4.9 ± 0.2	43.8 ± 13.5	48.5 ± 9.2	1.95 ± 0.04
In-25Sn	4.6 ± 0.2	5.4 ± 0.3	38.9 ± 5.6	38.2 ± 4.8	2.57 ± 0.15
In-30Sn	4.7 ± 0.1	5.8 ± 0.2	39.8 ± 1.2	46.4 ± 3.4	2.68 ± 0.17
In-35Sn	12.4 ± 0.3	16.3 ± 0.1	45.6 ± 6.1	31.4 ± 0.4	2.98 ± 0.07
In-40Sn	15.2 ± 0.2	20.1 ± 0.3	29.5 ± 4.4	20.0 ± 2.0	4.23 ± 0.15
In-50Sn	15.3 ± 0.7	17.2 ± 1.5	37.8 ± 7.7	35.6 ± 11.1	4.27 ± 0.37
In-60Sn	17.3 ± 0.2	21.5 ± 1.2	46.2 ± 12.6	38.4 ± 8.8	7.49 ± 0.29
In-80Sn	36.1 ± 0.3	37.8 ± 1.3	17.5 ± 2.1	16.8 ± 7.1	11.43 ± 0.34
Tin	15.8 ± 0.8	18.7 ± 0.7	49.4 ± 14.2	52.5 ± 5.2	8.13 ± 0.2

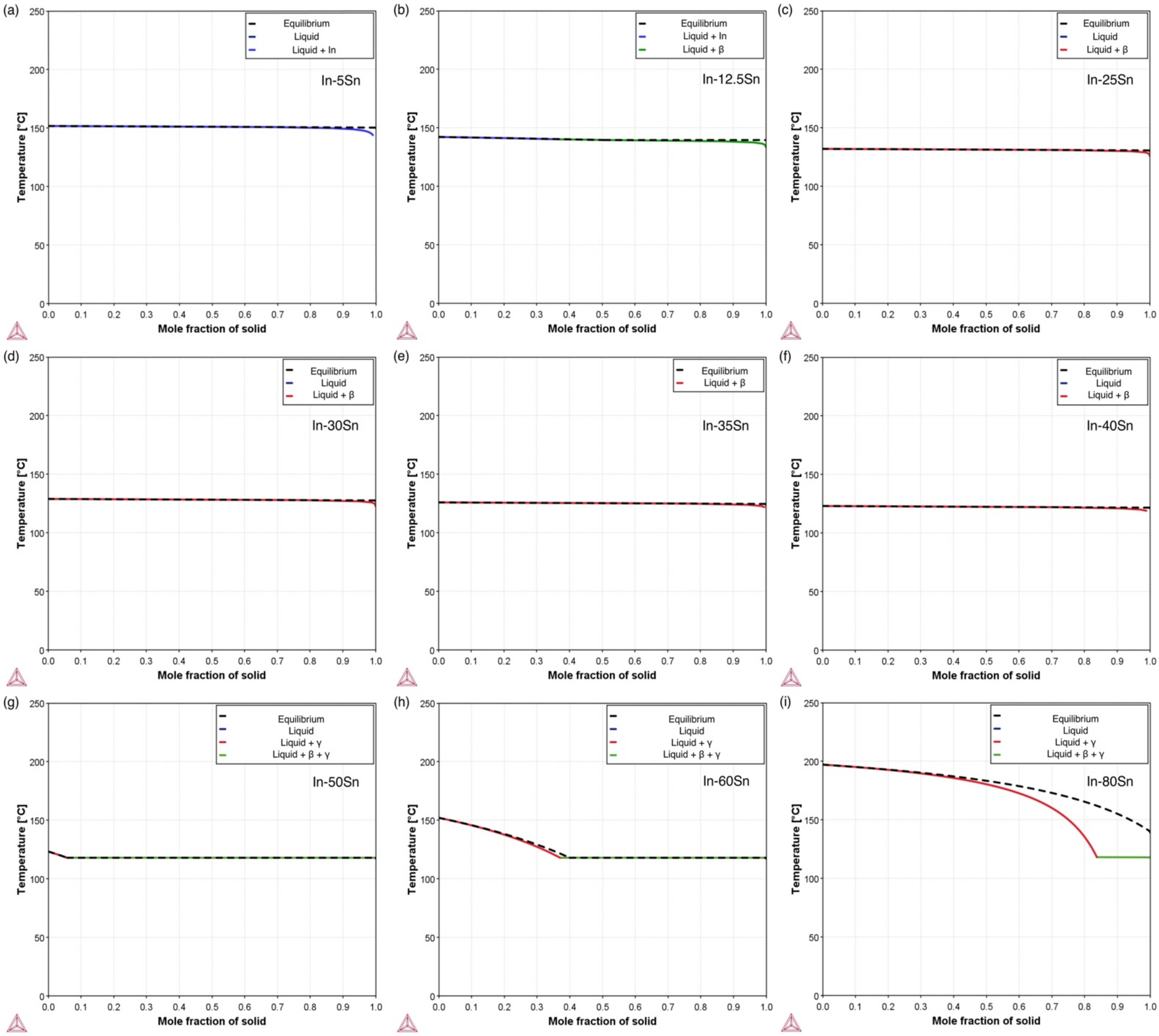

Figure S2. Volume fraction of phases in the In-Sn alloys during solidification calculated by Scheil equation (based on Thermo-Calc 2022a TCSLD4: Solder Alloys v4.1 database [1]) with increasing tin content from (**a**) to (**i**). The volume fraction of phases under the equilibrium condition is also plotted for comparison.

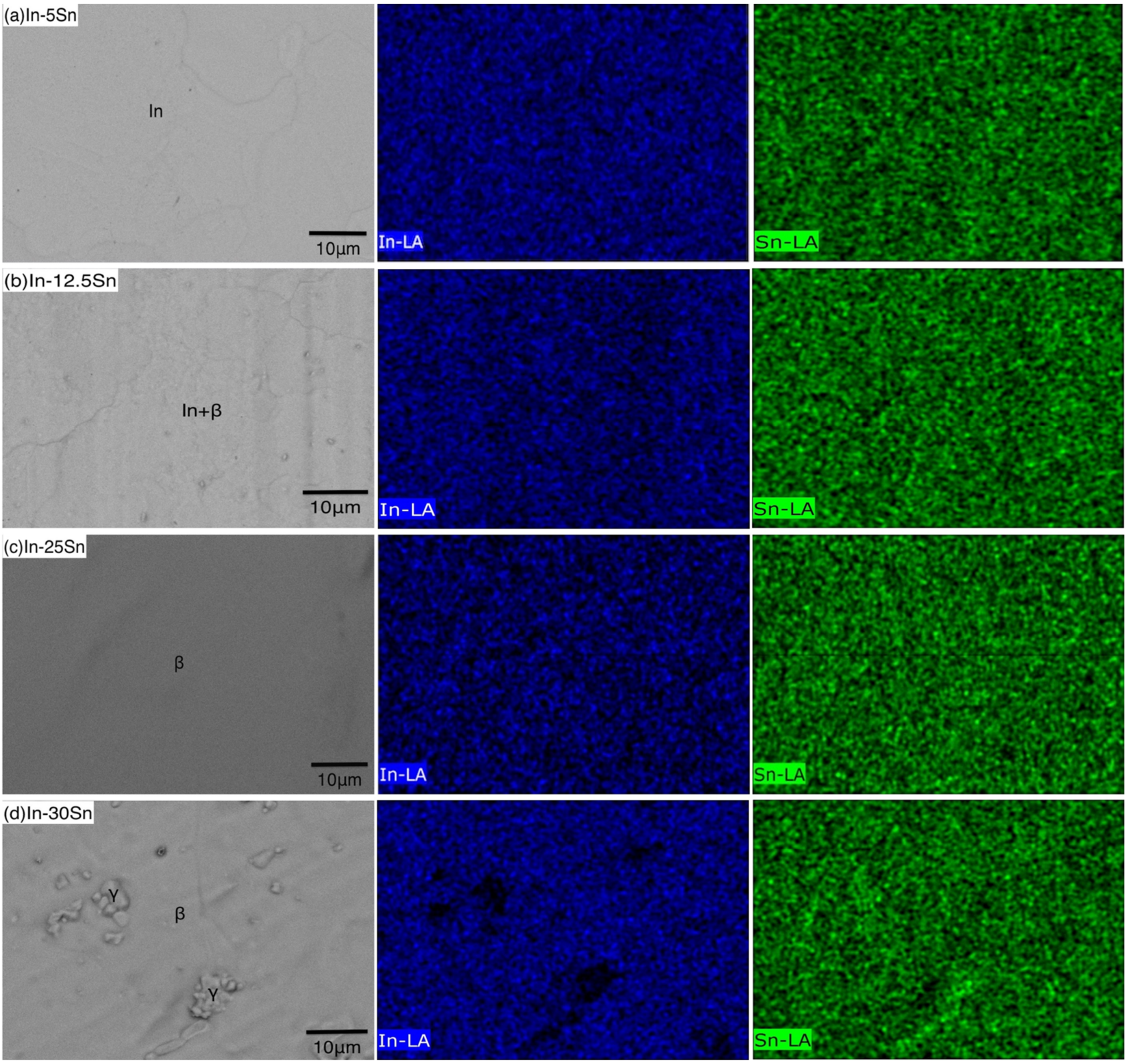
(a)In-5Sn
In
10μm
In-LA
Sn-LA
(b)In-12.5Sn
In+β
10μm
In-LA
Sn-LA
(c)In-25Sn
β
10μm
In-LA
Sn-LA
(d)In-30Sn
γ
β
γ
10μm
In-LA
Sn-LA

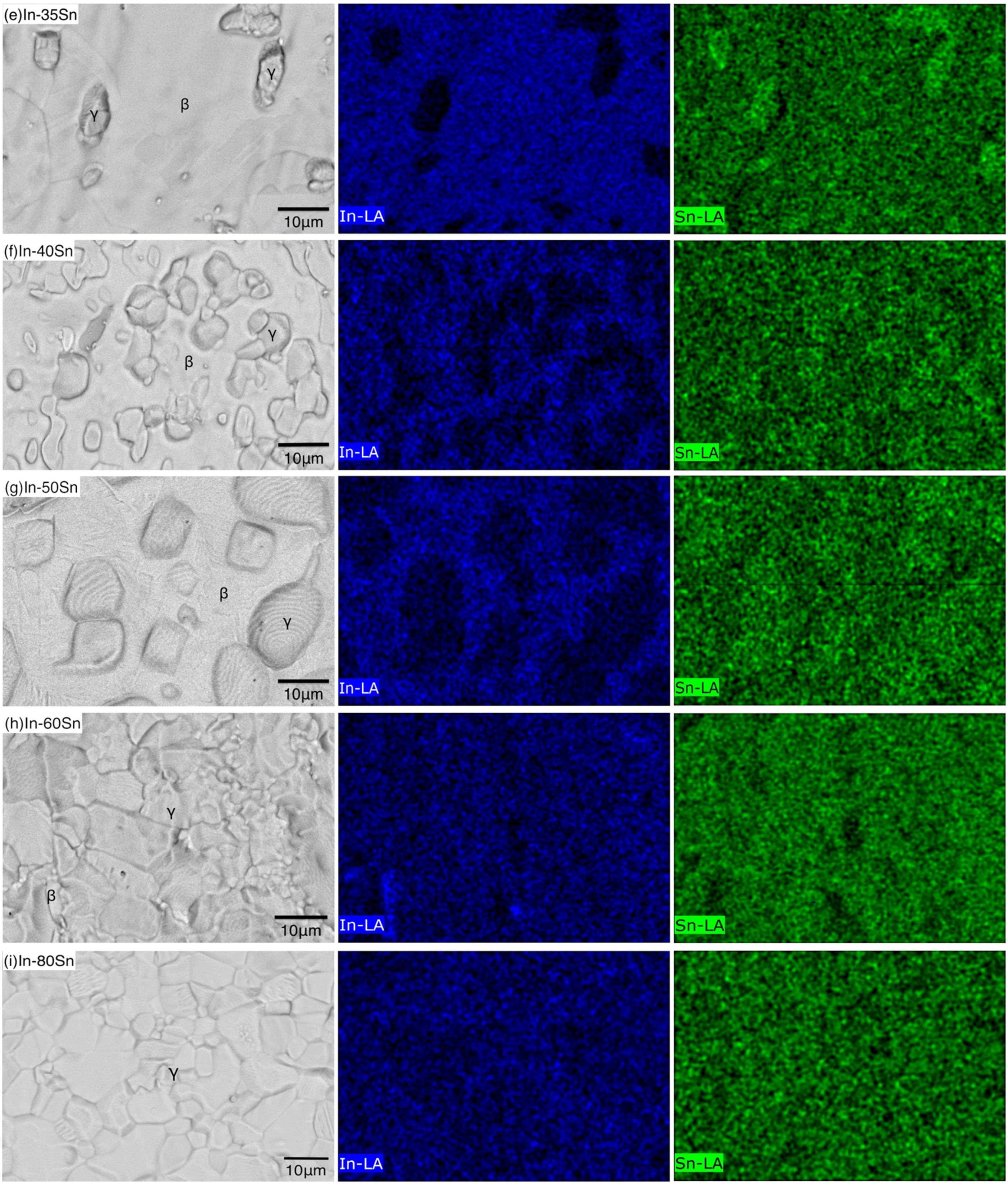

Figure S3. Microstructure and EDX mappings of In-Sn solder alloys with increasing tin content from (**a**) to (**i**).

Reference

1. Andersson, J.O.; Helander, T.; Höglund, L.; Shi, P.; Sundman, B. Thermo-Calc & DICTRA, computational tools for materials science. *Calphad* **2002**, *26*, 273–312. https://doi.org/10.1016/S0364-5916(02)00037-8.
